# Diagnostic Performance and Safety of Ultrasound‐Guided Core Needle Biopsy for Diagnosing Lymphoma: A Systematic Review and Meta‐Analysis

**DOI:** 10.1002/cam4.70414

**Published:** 2025-01-06

**Authors:** Yongmin Kwon, Min Kyoung Lee

**Affiliations:** ^1^ Department of Radiology, Yeouido St. Mary's Hospital, College of Medicine The Catholic University of Korea Seoul Republic of Korea

**Keywords:** core needle biopsy, lymphoma, meta‐analysis, systematic review, ultrasound

## Abstract

**Background:**

Lymphoma arises from transformed lymphoid cells. Although surgical excision biopsy is the standard diagnostic tool for patients with lymphoma, image‐guided fine‐needle aspiration (FNA) or core needle biopsy (CNB) is considered an alternative diagnostic option.

**Objective:**

To assess the diagnostic accuracy and safety of ultrasound (US)‐guided core needle biopsy (CNB) in patients with lymphoma.

**Methods:**

A systematic review and meta‐analysis were conducted. A literature search was performed up to January 1, 2024, using the Ovid‐MELIBE and EMBASE databases to identify studies focusing on US‐guided CNB in lymphoma patients. Relevant outcomes, including sensitivity, specificity, and complication rates, were extracted from the included studies. The Der‐Simonian‐Laird random‐effects model was applied to analyze the pooled data.

**Results:**

The pooled sensitivity of US‐guided CNB in lymphoma patients was 94% (95% CI = 89%–96%), and the specificity was 100% (95% CI = 94%–100%). The pooled complication rate was 1% (95% CI = 0%–3%), with self‐limiting complications being the most common.

**Conclusion:**

US‐guided CNB demonstrated high diagnostic accuracy and low complication rates in patients with lymphoma, supporting its use as an alternative diagnostic tool.

## 1 Introduction

Lymphoma, encompassing both Hodgkin lymphoma (HL) and non‐Hodgkin lymphoma (NHL), represents a neoplastic proliferation originating from transformed lymphoid cells [1]. The typical clinical manifestation often includes superficial or peripheral lymphadenopathy across various lymphoma subtypes [2]. Diagnosis relies on an amalgamation of morphological, immunophenotypic, and genetic characteristics, alongside clinical findings [3]. Critical to this evaluation is the assessment of lymph node architecture through tissue biopsy techniques such as fine‐needle aspiration (FNA), core needle biopsy (CNB), and surgical excision biopsy (SEB), with SEB being the preferred method due to its capacity for comprehensive tissue sampling [4]. However, recent advancements have endorsed less invasive techniques like image‐guided FNA and CNB for diagnosis and subclassification of lymphomas [5]. Numerous studies investigating the efficacy and safety of image‐guided CNB have utilized computed tomography (CT) and ultrasound (US) for imaging guidance [2]. Given the distinctive natural history of each lymphoma subtype, tailored management strategies underscore the importance of accurate subclassification [2, 6, 7]. Contemporary diagnostic methodologies enable precise classification even with limited tissue samples [8], with US‐guided CNB demonstrating efficacy in providing adequate tissue for architectural assessment and immunohistochemical analysis [9]. Multiple studies have confirmed the high diagnostic accuracy of US‐guided CNB in diagnosing and subclassifying lymphomas [10, 11, 12, 13, 14, 15], positioning it as a viable alternative diagnostic tool.

Although numerous studies have explored the outcomes of US‐guided CNB in patients with lymphoma, no systematic review or meta‐analysis has been undertaken to date. Hence, our objective was to conduct a systematic review and meta‐analysis to assess the diagnostic accuracy and safety of US‐guided CNB in this patient population.

## 2 Methods

This systematic review and meta‐analysis was conducted according to the Preferred Reporting Items for Systematic Reviews and Meta‐Analyses (PRISMA) guidelines (PROSPERO ID: CRD42022341231) [16].

### 2.1 Literature Search

We conducted a literature search using the Ovid‐MELIBE and EMBASE databases up to January 1, 2024, without date restrictions, to identify studies on the diagnostic performance of US‐guided CNB in patients with lymphoma. Search terms included (“lymphoma” OR “lymphoproliferative” OR “immunoproliferative” OR “malignant lymphoma”) AND (“ultrasonography” OR “US” OR “USG” OR “US‐guided” OR “sonogram” OR “ultrasound”) AND (“biopsy” OR “core needle” OR “needle”) AND (“excision” OR “dissection” OR “lymphadenectomy”). Selected articles underwent further examination to locate additional relevant studies. The literature search was independently conducted by one head and neck radiologist (M. K. L., 9 years of experience) and one training radiologist (M. K. L.). Discrepancies were resolved through consensus.

### 2.2 Inclusion and Exclusion Criteria

The inclusion criteria were as follows: (1) Population: patients diagnosed with histologically confirmed lymphoma; (2) Index test: cytopathology via US‐guided CNB; (3) Reference standard: diagnosis confirmed through excision followed by histopathological examination or clinical follow‐up; (4) Outcomes: diagnostic performance metrics (sensitivity, specificity), and complication rates; and (5) Study design: all observational (retrospective or prospective) original articles.

The exclusion criteria were s follows: (1) case reports, review articles, letters, editorials, conference abstracts, systematic reviews, and meta‐analyses; (2) insufficient data to compute diagnostic performance metrics for lymphoma based on true‐positive, true‐negative, false‐positive, and false‐negative rates; and (3) full‐text articles not available in English.

### 2.3 Data Extraction

The following information was extracted using a standardized form: (1) Study characteristics: first author, year of publication, affiliation, patient enrollment period, and study design (prospective/retrospective); (2) Demographic and clinical characteristics: numbers of total and male patients, mean age or range of included patients, and subtype of lymphoma; (3) Biopsy information: biopsy gun manufacturer, needle size, specimen number and size, biopsy location, number of biopsies performed by physician, and presence of immunohistochemistry (IHC); and (4) Outcomes: diagnostic performance of US‐guided CNB, including sensitivity, specificity, and complication rates.

### 2.4 Quality Assessment

Two reviewers (M. K. L. independently assessed the quality of the included studies using the revised Quality Assessment of Diagnostic Accuracy Studies‐2 (QUADAS‐2) tool, focusing on risk of bias and applicability. Disagreements were resolved through consensus.

### 2.5 Data Synthesis and Analysis

The primary outcome of this meta‐analysis was the diagnostic performance of US‐guided CNB in patients with lymphoma. Pooled sensitivity and specificity with 95% confidence intervals (CIs) were calculated using random‐effects modeling in individual studies. Summary receiver operating characteristic (SROC) curves with 95% CIs and predicted regions were graphically constructed. Heterogeneity was assessed using the Higgins *I* ^2^ statistic, with values ranging from 0% to 40% indicating insignificant heterogeneity, 30% to 60% indicating moderate heterogeneity, 50% to 90% indicating substantial heterogeneity, and 75% to 100% indicating considerable heterogeneity [17]. Deek's funnel plot was used to evaluate publication bias, and Deek's asymmetry test was used to assess its statistical significance [18]. Subgroup meta‐regression analyses were conducted to explore the sources of heterogeneity across studies, considering the following covariates: (1) needle size (< 18 gauge (G) vs. ≥ 18 G), (2) number of patients (< 100 vs. ≥ 100), and (3) biopsy location (cervical only vs. cervical and other regions). For meta‐analytic pooling of the complication rate, the inverse variance method was used to calculate weights, and the Der‐Simonian‐Laird random‐effects model was used to calculate 95% CIs [19]. Statistical analyses were performed using STATA version 18.0 (StataCorp, College Station, TX, USA), with *p* values < 0.05 considered statistically significant.

## 3 Results

### 3.1 Literature Search

**FIGURE 1:**
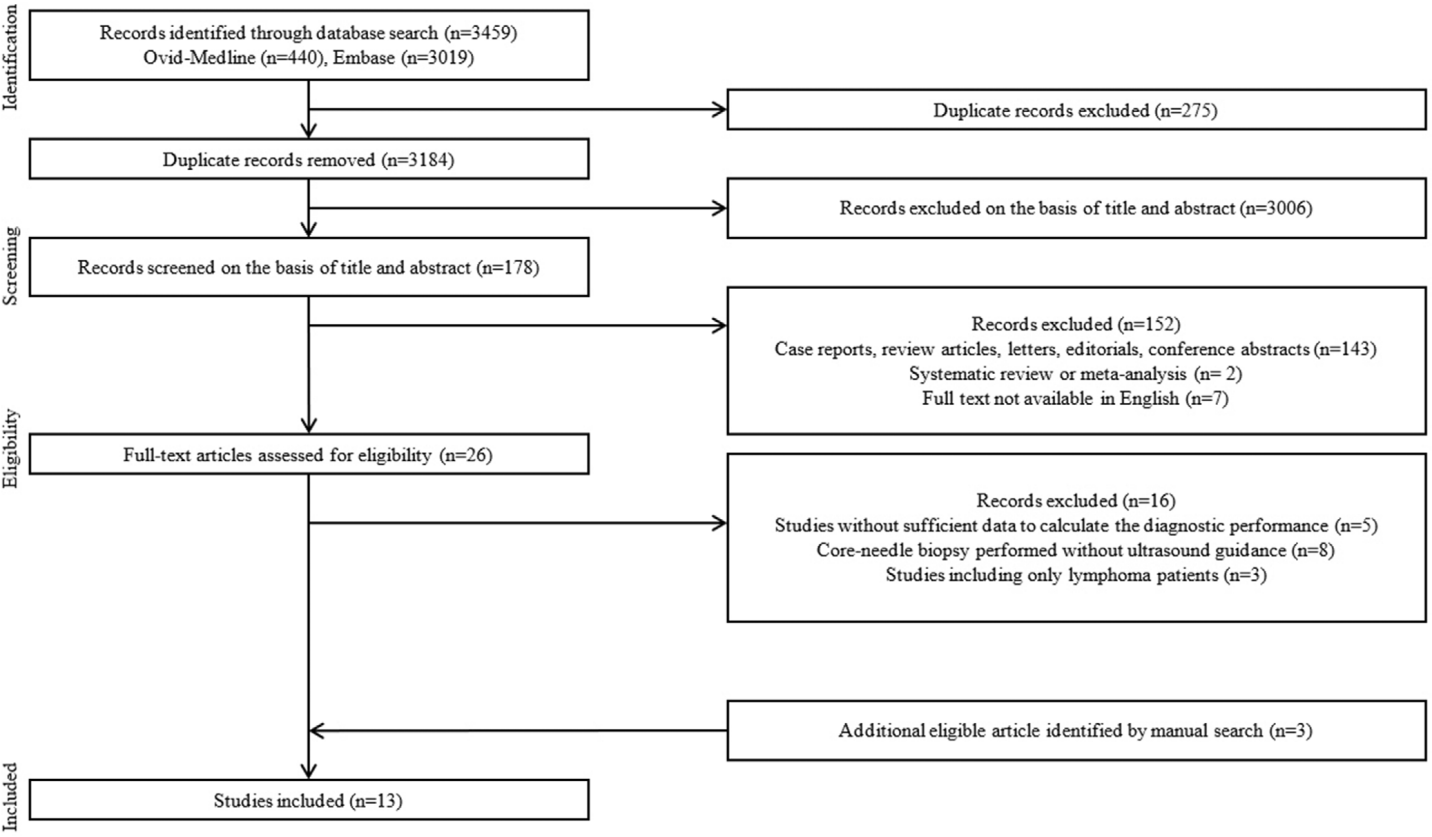
PRISMA flowchart of selecting process of eligible studies.

Figure 1 shows a flow diagram describing the study selection process. Initially, 3459 papers were identified, and 275 duplicate studies were removed. Subsequently, 3184 papers underwent screening on the basis of titles and abstracts, resulting in 178 papers for further evaluation of their eligibility. Among these, the first 152 studies were excluded for being case reports, review articles, letters, editorials, conference abstracts (*n* = 143), systematic reviews, or meta‐analyses (*n* = 2), or if the full text was unavailable in English (*n* = 7). A full‐text review followed, excluding 16 studies due to insufficient data to calculate diagnostic performance (*n* = 5) [20, 21, 22, 23, 24], or utilizing CNB methods other than US‐guided CNB (*n* = 8) [25, 26, 27, 28, 29, 30, 31, 32], or covering only patients with lymphoma (*n* = 3) [5, 33, 34]. Three additional articles were identified as eligible through a manual search [9, 10, 35]. Finally, 13 studies were included in this systematic review and meta‐analysis [9, 10, 11, 12, 13, 14, 15, 35, 36, 37, 38, 39, 40].

### 3.2 Characteristics of the Included Studies

The demographic characteristics of the 13 included studies are shown in Table 1 [9, 10, 11, 12, 13, 14, 15, 35, 36, 37, 38, 39, 40]. Two studies were prospectively designed [14, 39], whereas the other 11 were retrospectively designed [9, 10, 11, 12, 13, 15, 35, 36, 37, 38, 40]. The number of included participants ranged from 24 to 735, with the proportion of male patients ranging from 8% to 64% in nine studies [9, 11, 12, 14, 15, 35, 38, 39, 40]. excluding four studies [10, 13, 36, 37], in which data were not available. The mean or median age of the included patients ranged from 38.0 to 61.1 years in 10 studies [9, 11, 12, 13, 14, 15, 35, 36, 38, 40], except for three studies [10, 37, 39] because of a lack of precise age information. Among the studies that covered lymphoma subtypes [9, 10, 11, 12, 13, 14, 15, 35, 38, 40], more than half resulted in a larger proportion of NHL cases, as proven by US‐guided CNB, than HL [9, 10, 11, 13, 14, 15, 40]. Subtype information was not mentioned in any of the three studies [36, 37, 39]. Table 2 presents the biopsy data from the included studies. Among the included studies, six studies [13, 14, 35, 36, 37, 39] performed US‐guided CNB only in the cervical area, including the cervical node, non‐nodal cervical or facial mass, parotid gland, and submandibular gland, whereas seven studies [9, 10, 11, 12, 15, 38, 40] included biopsy sites in the cervical area and other regions, including the axilla, mediastinum, groin, breast, flank, and abdomen. Most of the included studies performed IHC using US‐guided CNB specimen [9, 10, 11, 12, 13, 14, 15, 35, 38, 39, 40], whereas two studies did not evaluate IHC [36, 37].

**TABLE 1:** Demographics and clinical characteristics of the included studies.

First author (year of publication)	Affiliation	Patient enrollment period	Study design	No. of participants	Male (%)	Mean age (range)	Subtype of lymphoma
Adeel (2021)	Royal Hallamshire Hospital, Sheffield Teaching Hospitals, Sheffield, UK	May 2017–Apr 2019	Retrospective	287	NA	58.1	NA
Allin (2017)	Guy's and Saint Thomas' NHS Foundation Trust, London, UK	Dec 2013–Apr 2015	Retrospective	70	NA	NA	NA
Baer^a^ (2021)	Johns Hopkins University School of Medicine, Baltimore, US	Jul 2009–Aug 2018	Retrospective	24	2 (8%)	NA (18–74)	MZL of MALT
Cohen (2021)	University College London Hospitals NHS Foundation Trust, London, UK	2016–2018	Retrospective	512	NA	NA	HL (17.3%) (cHL, NLPHL) NHL (82.7%) (DLBCL, FL, CLL/SLL, MCL, NMZL, HGBL PTCL, etc.)
Elhamdoust (2020)	Golestan Hospital, Ahvaz Jundishapur University of Medicine, Ahvaz, Iran	2019	Retrospective	40	18 (45%)	49.4	HL (56.5%), NHL (43.5%)
Groneck (2016)	Klinik I für Innere Medizin, Universitätsklinik Köln, Köln, Germany	NA	Retrospective	138	88 (64%)	NA (17–82)	HL (23.3%) (cHL, NLPHL) NHL (76.7%) (DLBCL, FL, etc.)
Howlett (2006)	Eastbourne District General Hospital, Kings Drive, Eastbourne, UK	NA (over 3.5 years period)	Prospective	82	46 (56%)	NA (24–93)	NA
Kiliçarslan (2017)	University of Ankara Yıldırım Beyazıt School of Medicine, Ankara, Turkey	2010–2016	Retrospective	60	37 (62%)	50.8 (19–74)	HL (53%) NHL (47%) (DLBCL, MCL, CLL)
Kim (2007)	Sungkyunkwan University School of Medicine, Kangbuk Samsung Hospital, Seoul, Korea	Mar 2000–Sep 2005	Retrospective	155	NA	38 (13–82)	HL (25%) NHL (75%) (DLBCL, Burkitt lymphoma, ALCL, MCL, Precursor B‐cell lymphoma)
Nguyen (2014)	Harbor‐UCLA Medical Center, California, USA	Apr 2008–Jan 2014	Retrospective	71	31 (44%)	43.1 (18–71)	HL (32%) (cHL, NLPHL) NHL (68%) (FL, DLBCL, THRBCL, NMZL, Plasmablastic lymphoma, etc.)
Pfeiffer (2009)	University of Freiburg, Freiburg, Germany	Apr 2003–Oct 2007	Prospective	45	27 (60%)	61.1 (21–91)	HL (7.7%) (cHL) NHL (92.3%) (DLBCL, FL, MCL, Marginal zone lymphoma, etc.)
Pugliese (2017)	Department of Clinical Medicine and Surgery, Federico II University Medical School, Naples, Italy	Jan 2009– Dec 2015	Retrospective	185	86 (47%)	NA (17–76)	HL (30.1%) (cHL, NLPHL) NHL (69.9%) (DLBL, FL, CLL/SLL, MCL, NMZL, ALCL, PTCL, etc.)
Wilczynski^b^, ^c^ (2020)	University Hospital Marburg und Giessen, Marburg, Germany	Jan 2006–Jun 2015	Retrospective	735	430 (59%)	60 (11–90)	HL (17.8%) NHL (82.2%) (B‐cell lymphoma, T‐cell lymphoma, posttransplantation lymphoma)

Abbreviations: AILD, angioimmunoblastic T‐NHL; ALCL, anaplastic large cell lymphoma; ALL, acute lymphocytic leukemia; cHL, classic Hodgkin's lymphoma; CLL, chronic lymphocytic leukemia; DLBCL, diffuse large cell B‐cell lymphoma; FL, follicular lymphoma; HGBL, high‐grade B‐cell lymphoma; HL, Hodgkin's lymphoma; MCL, mantle cell lymphoma; NHL, non‐Hodgkin's lymphoma; NLPHL, nodular lymphocyte predominant Hodgkin's lymphoma; NMZL, nodal marginal zone lymphoma; PTCL, peripheral T‐cell lymphoma; SLL, small lymphocytic lymphoma; THRBCL, T‐cell‐rich large B‐cell lymphoma.

^a^ Only including patients with Sjögren's syndrome (SS).

^b^ Including posttransplantation lymphoma as NHL.

^c^ Pooled sensitivity and specificity calculated by number of cases.

**TABLE 2:** Biopsy information of the included studies.

First author (year of publication)	Biopsy gun manufacturer	Needle size	Specimen number	Specimen size	Biopsy location	No. of biopsy performed physician	Immunohistochemistry
Adeel (2021)	NA (Temno biopsy needle)	16 G or 18 G	Average 2–3	NA	Cervical node, parotid gland, neck‐lump (non‐nodal), submandibular gland	1	NA
Allin (2017)	Carefusion (Temno biopsy needle)	16 G	NA	NA	Neck	NA	NA
Baer (2021)	INRAD	18 G	1–4 (2.29 ± 0.66 per gland)	NA	Submandibular gland, parotid gland	NA (> 1)	Y
Cohen (2021)	Argon medicine (Biopince), Cook (Quickcore)	16 G (major), 18 G	1–4 (median 3 core)	1 cm	Lymph nodes (cervical, axillary, inguinal), extra‐nodal	NA (> 1)	Y
Elhamdoust (2020)	NA	NA	NA	NA	Superficial (neck, axillary, inguinal, breast, vertical muscle, waist), abdominal	NA	Y
Groneck (2016)	NA	14 G, 16 G, 18 G	NA	NA	Cervical, supraclavicular, axillary, inguinal	NA	Y
Howlett (2006)	Franklin Bard (Biopty gun)	18 or 20 G	NA (mean 2)	NA	Neck (thyroid and salivary gland excluded)	1	Y
Kiliçarslan (2017)	NA	NA	NA	NA	Cervical, axillary, supraclavicular, abdominal, submandibular, inguinal	NA	Y
Kim (2007)	Manan (Pro‐Mag 2.2) TSK Laboratory (Stericut)	16 G	2–4 (mean 2.5)	Throw 2.2 cm	Neck	2	Y
Nguyen (2014)	CareFusion (Achieve core biopsy needle)	14 G	8–10	NA	Cervical, supaclavicular, axillary, inguinal	1	Y
Pfeiffer (2009)	Bard (Bard Magnum)	12 G–16 G	1–4 (mean 2.16)	NA	Cervico‐facial mass	NA	Y
Pugliese (2017)	Biomol HS‐Hospital (Modified Menghini needle with automatic aspiration)	16 G	1–4 (median 2)	15–70 mm	Cervical, axillary, mediastinum, inguinal, abdominal	2	Y
Wilczynski (2020)	NA	18 G	1–4	9 mm, 19 mm, 29 mm	Peripheral (cervical, supraclavicular, inguinal, axillary), abdominal	1	Y

Abbreviations: NA, not applicable; Y, yes.

### 3.3 Quality Assessment

**FIGURE 2:**
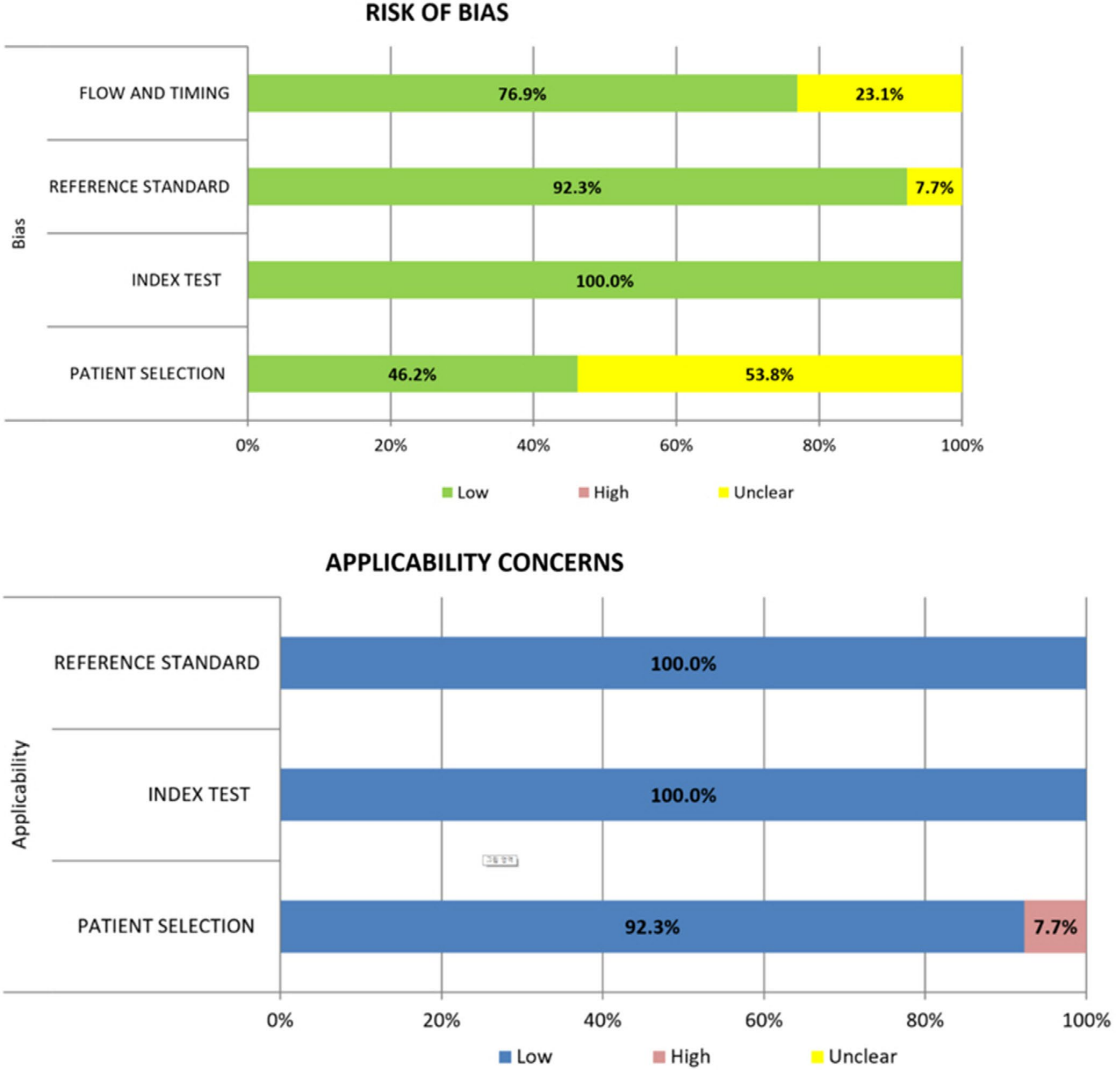
Quality assessment of the included studies according to the QUADAS‐2 criteria.

Five studies fulfilled all seven domains, four fulfilled six domains, and four fulfilled five domains (Figure 2). Six studies [9, 14, 36, 38, 39, 40] had a low risk of bias in patient selection, whereas the other seven studies had an unclear risk of bias [10, 11, 12, 13, 15, 35, 37]. All studies showed a low risk of bias in the index test domain (Figure 2). One study had an unclear risk of bias in the reference standard domain because there were two patients whose biopsy results were unclassifiable even after SEB [38]. Other 12 studies had a low‐risk bias in the same domain [9, 10, 11, 12, 13, 14, 15, 35, 36, 37, 39, 40]. Three studies resulted in an unclear risk of bias in the flow and timing domains because the specific method for secondary diagnosis was not stated [10, 11, 37], whereas the other 10 studies showed low‐risk bias [9, 12, 13, 14, 15, 35, 36, 38, 39, 40]. Nearly all the studies were categorized as having low concerns regarding applicability in the patient selection, index test, and reference standard domains. Only one study was of high concern for applicability in patient selection because it included only patients with Sjogren's syndrome (SS) [35].

### 3.4 Pooled Estimation of Diagnostic Performance

**FIGURE 3:**
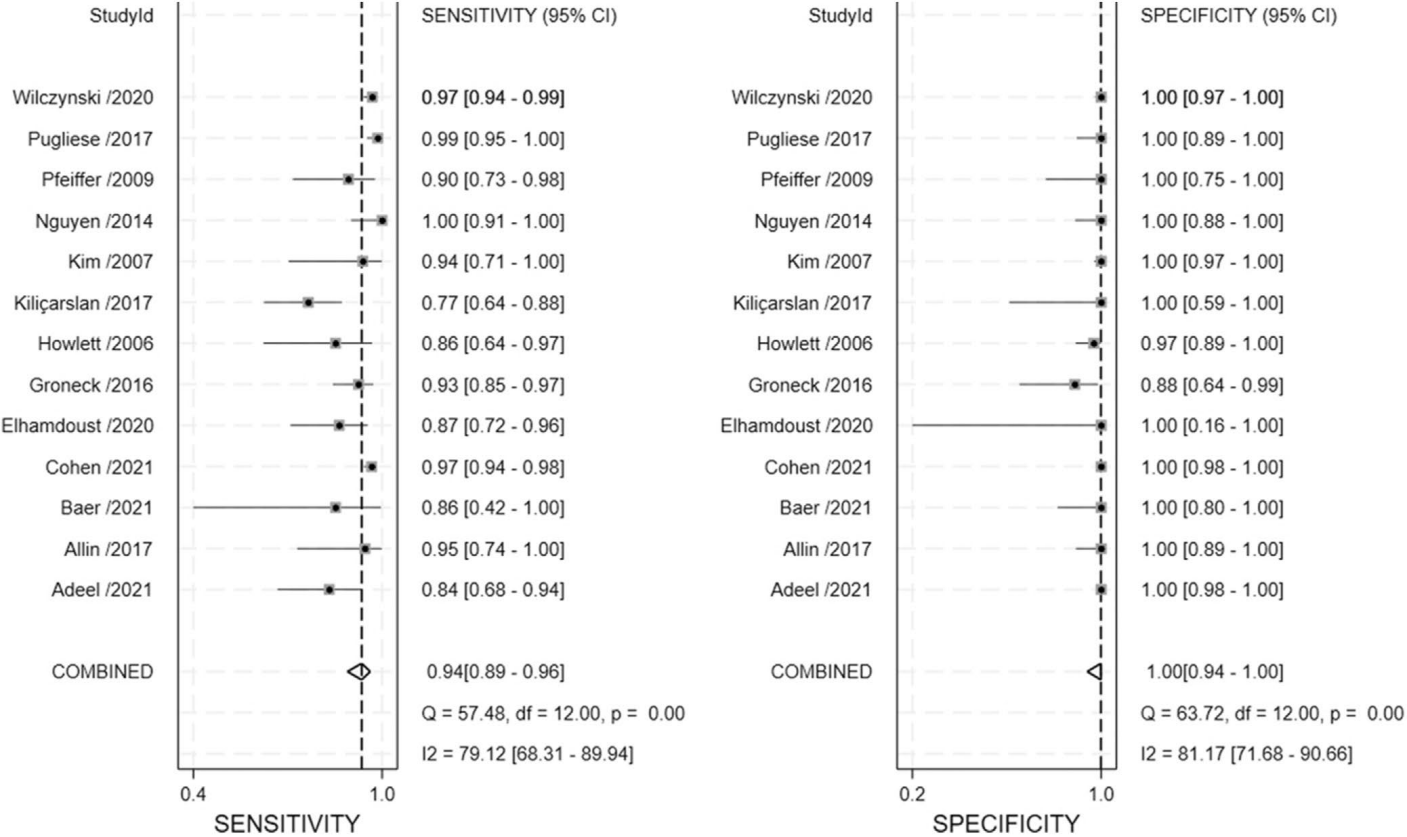
Coupled plots of pooled sensitivity and specificity of US‐guided CNB in diagnosis of patients with lymphoma. Horizontal lines presents 95% CIs of each study. CNB, core needle biopsy; CI, confidence interval; US, ultrasound.

The diagnostic performance, represented by sensitivity and specificity, is shown in Figure 3 as an illustration of the coupled forest plots of the pooled data. Among the included studies evaluating the diagnostic performance of US‐guided CNB in patients with lymphoma, the pooled sensitivity was 94% (95% CI: 89%–96%) and specificity was 100% (95% CI: 94%–100%). Considerable heterogeneity was noted in sensitivity (*I* ^2^ = 79.12%, *p* < 0.001) and specificity (*I* ^2^ = 81.17%, *p* < 0.001). The SROC curve showed a large difference between the areas with 95% confidence and predicted regions (Figure S1). There was significant publication bias among the included studies on funnel plots (Figure S2) and Egger's test (*p* < 0.001).

**FIGURE 4:**
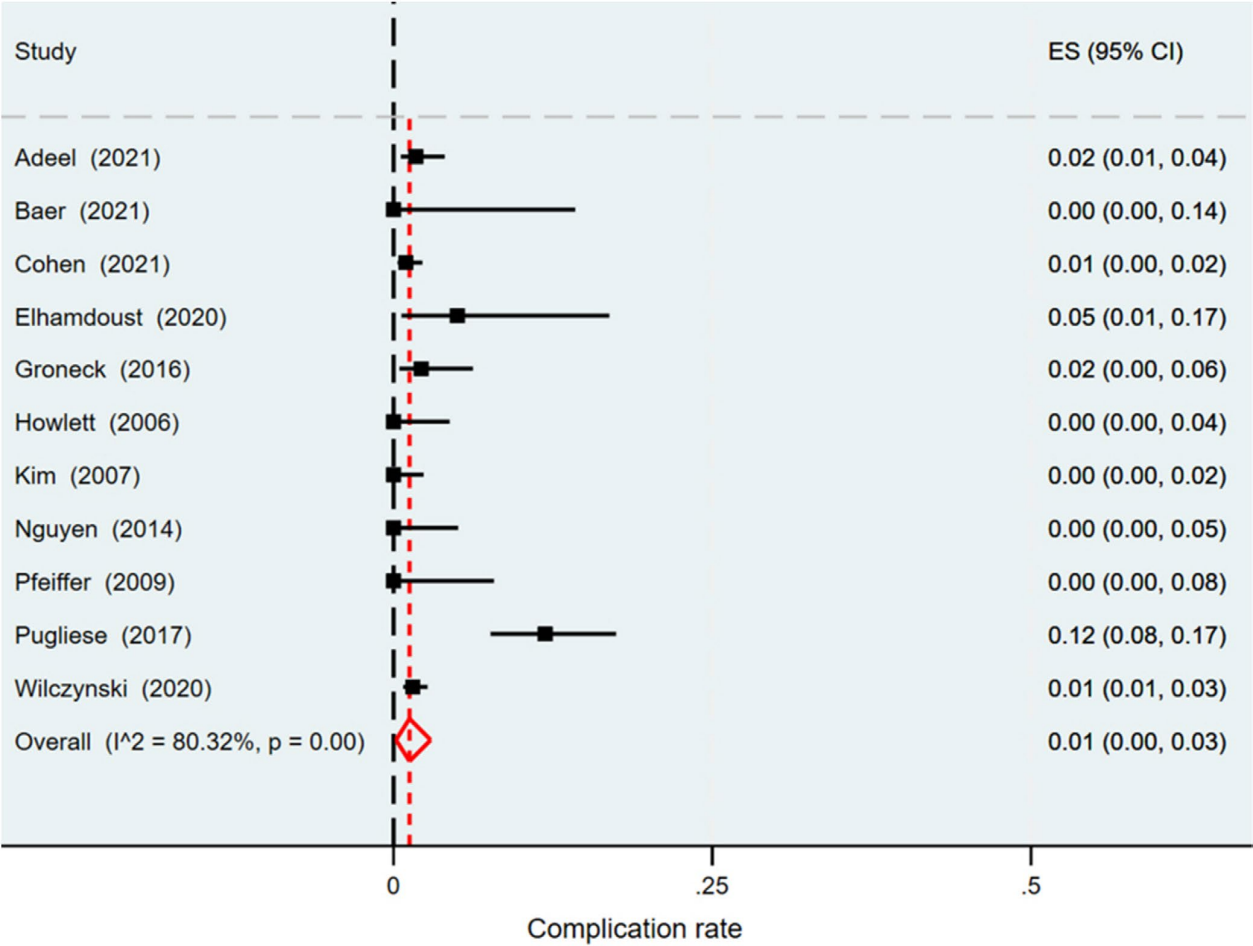
Forest plot of pooled complication rates of US‐guided CNB. Horizontal lines presents 95% CIs of each study. CNB, core needle biopsy; CI, confidence interval; US, ultrasound.

The pooled complication rate of US‐guided CNB is summarized in Figure 4, and two studies were excluded because of the absence of information [12, 37]. The pooled complication rate was 1% (95% CI: 0%–3%), with considerable heterogeneity (*I* ^2=^80.32%, *p* < 0.001). Most complications were self‐limiting; therefore, they were managed conservatively and were not associated with long‐term morbidity. The most common complications were minor bleeding and hematoma, followed by pain (Table S1). Other complications included bruising, vasovagal attack, transient facial weakness related to local anesthesia, self‐limiting lymph fistula, and transient hypoesthesia of the trigeminal nerve. Only one case was reported as a major complication [15], in which active bleeding occurred after biopsy of a cervical lymph node with a venous malformation inside it.

### 3.5 Subgroup Meta‐Regression Analysis

The subgroup meta‐regression analysis results are outlined in Table 3. We assessed the included studies using three covariates (needle size, number of patients, and biopsy location) to explore heterogeneity sources. Studies utilizing needles with larger circumferences (smaller gauge, < 18 G) for US‐guided CNB exhibited significantly higher pooled sensitivity (97% vs. 94%, *p* < 0.001) and specificity (100% vs. 95%, *p* < 0.001) compared with those using smaller circumferences (larger gauge, ≥ 18 G). There were no significant differences in diagnostic performance observed for other variables, including the number of patients and biopsy location.

**TABLE 3:** Subgroup meta‐regression analyses for identifying heterogeneity.

Covariate	No. of studies	Sensitivity	Specificity	*p*	*I* ^2^
Needle size (circumferential)				< 0.001	89
< 18G	5	97	100		
≥ 18G	6	94	100		
Number of patients				0.23	31
< 100	7	90	100		
≥ 100	6	96	100		
Biopsy location				0.38	0
Only cervical	6	90	100		
Cervical and other regions	7	95	100		

## 4 Discussion

This systematic review and meta‐analysis evaluated published studies examining the efficacy and safety of US‐guided CNB for diagnosing lymphoma. Our findings indicate excellent diagnostic performance of US‐guided CNB, with a pooled sensitivity of 94% (95% CI: 89%–96%), specificity of 100% (95% CI: 94%–100%), and an area under the SROC curve of 99% (95% CI: 98%–100%). Complications associated with US‐guided CNB occurred at a pooled rate of 1%, predominantly minor. Traditionally, lymphoma diagnosis relies on SEB, but recent evidence suggests US‐guided CNB as a viable alternative. Notably, this study represents the first systematic review and meta‐analysis investigating the diagnostic accuracy and safety of US‐guided CNB in patients with lymphoma. Our findings strongly support the potential of US‐guided CNB as an alternative diagnostic approach to SEB for lymphoma diagnosis.

Recently, there have been several papers [10, 11, 12, 13, 14, 15, 38] on the diagnostic performance of US‐guided CNB in patients with lymphoma, such as those included in this study. Although most published studies have not directly compared US‐guided CNB and SEB for the diagnosis of lymphoma, they showed good diagnostic performance. A recent multicenter French study involving 32,285 cases compared US‐guided CNB and SEB for lymphoma diagnosis [41]. They found SEB to have significantly higher diagnostic performance, with 98.1% sensitivity, whereas US‐guided CNB exhibited lower sensitivity at 92.3% (*p* < 0.0001). Another comparative study yielded similar results, showing SEB with higher diagnostic performance at 98.8% sensitivity compared with US‐guided CNB at 95.9% sensitivity (*p* = 0.049) [42]. Although US‐guided CNB showed a lower sensitivity than SEB for lymphoma diagnosis, the authors mentioned that considering clinical information and IHC data could improve the diagnostic performance of US‐guided CNB. In addition, a large‐scale, multi‐institutional study evaluated the diagnostic yield of small volume biopsy (SVB), which comprises FNA with or without CNB, across common clinical indications throughout the disease course of follicular lymphoma [43, 44, 45]. Although SVBs may not always allow for complete classification, they usually provide sufficient information for clinical decision‐making, particularly in cases of recurrent or transformed lymphoma. An initial investigation found that for follicular patients with lymphoma with suspected transformation, the time to diagnosis was comparable between initial biopsy methods (SVB or SEB), suggesting that starting with SVB is unlikely to delay diagnosis or treatment [43]. Notably, the subclassification rate increased with CNB at the time of initial follicular lymphoma diagnosis [44]. A follow‐up study reviewed diagnostic discrepancies between the initial SVB and subsequent biopsy performed within 3 months [45]. In this cohort, the initial SVB demonstrated 70% sensitivity for lymphoma diagnosis, with 7% yielding nondiagnostic results. Across all disease stages, SVB showed 100% specificity, with no instances of overdiagnosis or downgrading from malignant to benign. Therefore, the authors suggested that US‐guided CNB could serve as an alternative tool for lymphoma diagnosis. Evaluating the safety of a technique is crucial for assessing the clinical implications of diagnostic tools. A recent study reported a 5.9% complication rate for SEB, primarily consisting of minor complications (84.2%) [46]. Other studies also reported complication rates ranging from 2.5% to 6.5% [25, 47]. Our study demonstrated a lower pooled complication rate of 1%.

The present meta‐analysis showed good performance, with 94% pooled sensitivity and 100% pooled specificity for US‐guided CNB in the diagnosis of lymphoma. In lymphoma diagnosis, IHC results are important for developing a treatment strategy and can be an important factor for improving the diagnostic performance of US‐guided CNB [2, 6, 7, 42]. Whether the specimen obtained through US‐guided CNB is sufficient to evaluate IHC is the most important question for evaluating diagnostic performance. Most of the included studies performed IHC using US‐guided CNB specimens. Our study shows that US‐guided CNB provides enough tissue for lymphoma diagnosis. Elhamdoust et al. showed the supporting results that IHC results can improve the diagnostic performance of US‐guided CNB and that US‐guided CNB provides sufficient tissue for evaluating IHC [38]. Therefore, US‐guided CNB provides an adequate amount of tissue for lymphoma subtyping using IHC, which can contribute to the development of appropriate treatment plans.

Although US‐guided CNB has shown good diagnostic performance and safety in the diagnosis of lymphoma, several issues have been considered. In particular, the diagnostic performance differed according to the lymphoma subtype because it may not be enough to give a final diagnosis solely by US‐guided CNB in HL patients, rather than NHL patients [48]. Groneck et al. showed that HL had a higher false‐negative rate on US‐guided CNB than NHL [11]. All false‐negative US‐guided CNB results for patients with HL were clearly detected with a secondary biopsy, such as SEB or biopsy from other sites, or confirmed by an expert review. The sensitivity for clinically conclusive histopathological diagnosis of US‐guided CNB specimens of lymph nodes or tumors was 96.7% for NHL and only 66.0% for HL. Other studies have also shown similar results; the misdetection rate of HL was higher than that of NHL [49, 50]. The relatively poor diagnostic performance of US‐guided CNB in patients with HL may be due to its complex architecture [11]. Therefore, we recommend repeating US‐guided CNB, consulting an expert review, or performing SEB in patients with HL for an accurate diagnosis.

Moreover, there is no standard US‐guided CNB technique for lymphoma diagnosis. In our subgroup analysis, needle with larger circumferential (smaller gauge, < 18 G) resulted in a better diagnostic performance with a significantly higher pooled sensitivity and similar pooled specificity (< 18 G vs. ≥ 18 G; 97% and 100% vs. 94% and 100%, *p* < 0.001). These results suggest that more histopathologic information can be obtained from larger specimens, but because no study has addressed the details of needle size in each biopsy case, we could conclude that no definite statistical evidence has been achieved for the advantage of a needle with a smaller gauge. Groneck et al. suggested a slightly better, yet not significant, outcome with the use of 14 G needles than with 16 G and 18 G needles, concluding that needle size is not crucial [11]. However, another study recommended the use of not too small cutting needles (≥ 16 G) [14]. Both studies agree that acquiring multiple cores from different areas of the lymph nodes is important for obtaining good sampling results. Accordingly, to improve the quality of biopsy samples, biopsies should be performed more than once in various regions of the target lymph node using a larger cutting needle, if necessary. The debate between CNB and SEB in lymphoma diagnosis remains ongoing. Although 100% specificity in CNB is commendable, for SEB it may reflect the potential for unnecessary procedures in some cases [51]. However, less invasive techniques such as FNA and CNB, especially when combined with ancillary studies, offer safe, rapid, and accurate diagnoses in most cases [52]. Each method, including SEB, has its own strengths and limitations. One study advice caution against the routine use of CNB, favoring SEB in appropriate cases due to potential diagnostic pitfalls in certain lymphoma subtypes [51]. On the contrary, another study highlights the practicality and accuracy of less invasive methods, particularly when enhanced by advanced ancillary techniques [52]. Ultimately, the choice between these approaches should be guided by individual patient factors, suspected pathology, and available expertise. Although less invasive methods have shown promising results, SEB remains an important option in more complex cases. This ongoing debate underscores the need for continued research and a multidisciplinary approach in lymphoma diagnostics.

Our study had a few limitations. First, among the 13 articles, very few included a direct comparison of US‐guided CNB with SEB in the diagnosis of lymphoma [12, 13, 38]. Considering the difficulty to proceed meta‐analysis on this subject, if related future papers are more published, further study should be performed to investigate and approve the diagnostic performance of US‐guided CNB in patients with lymphoma, directly compared with SEB. Second, the number of included studies providing details of the specimen number (number of US‐guided CNB passes) [9, 10, 13, 14, 15, 35, 40] and the size of the biopsied lymph nodes [9, 10, 13, 15] were too small for subgroup analysis. Due to a lack of information, the reported specimen number or size in the included studies [9, 10, 13, 14, 15, 35, 40] is limited, making it difficult to draw conclusions about the relationship between the mentioned biopsy information and the diagnostic performance of US‐guided CNB. Third, a challenge lies in conducting a detailed analysis of the subtypes of NHL. Although we listed the subtypes mentioned in the included studies, extracting data specific to each subtype was difficult, making it impractical to perform a thorough subgroup analysis. Similarly, although the subtype of HL was referenced in several reviewed papers, the available data did not allow for in‐depth exploration of these subtypes. This limitation may have influenced the ability to fully assess the nuanced differences in sensitivity and specificity across lymphoma subtypes. Finally, due to limited information provided in the studies we reviewed, it was challenging to conduct a comparative analysis of diagnostic accuracy before and after the application of flow cytometry or molecular studies. Among the five mentioning the performance of molecular biological studies [9, 10, 11, 14, 35], studies reported that CNB provides sufficient material for molecular analyses [9, 10, 11]. Moreover, through literature search, notable studies have been identified that address the importance of flow cytometry in needle biopsy of lymphoma, both in diagnosis and subclassification [53, 54, 55, 56, 57].

In conclusion, this review suggests that US‐guided CNB shows promising diagnostic performance and low complication rates in the diagnosis of lymphoma. Although the results indicate that US‐guided CNB may be a useful diagnostic tool for patients with lymphoma, further research is needed to confirm its effectiveness as an alternative to other diagnostic methods.

## Author Contributions

**Yongmin Kwon:** data curation (equal), formal analysis (equal), investigation (equal), methodology (equal), writing – original draft (equal). **Min Kyoung Lee:** conceptualization (lead), data curation (equal), formal analysis (equal), funding acquisition (lead), investigation (equal), methodology (lead), project administration (lead), resources (lead), software (lead), supervision (lead), validation (lead), visualization (lead), writing – original draft (equal), writing – review and editing (lead).

## Conflicts of Interest

The authors declare no conflicts of interest.

## Supporting information


**Figure S1.** Summary receiver operating characteristics (SROC) curves of US‐guided CNB for diagnosis in patients with lymphoma.

**Figure S2.** Deek’s funnel plot asymmetry test of 13 included studies.

**Table S1.** Outcomes: complications of USCNB.

## Data Availability

Data are openly available in a public repository that issues datasets with DOIs.
