# Supplementary figures and images for: Diagnostic Performance and Safety of Ultrasound‐Guided Core Needle Biopsy for Diagnosing Lymphoma: A Systematic Review and Meta‐Analysis
Source: Cancer Med. 2025 Jan 6;14(1):e70414. doi: 10.1002/cam4.70414 (PMC11702417; doi:10.1002/cam4.70414)

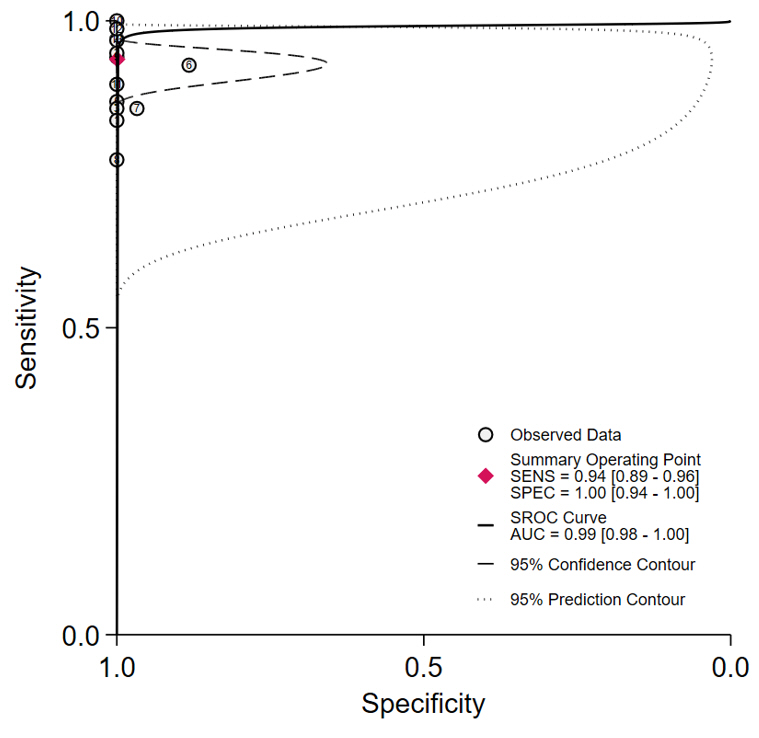

Supplement: Supplementary file 1 — Figure S1. Summary receiver operating characteristics (SROC) curves of US‐guided CNB for diagnosis in patients with lymphoma. [file CAM4-14-e70414-s002.jpg]

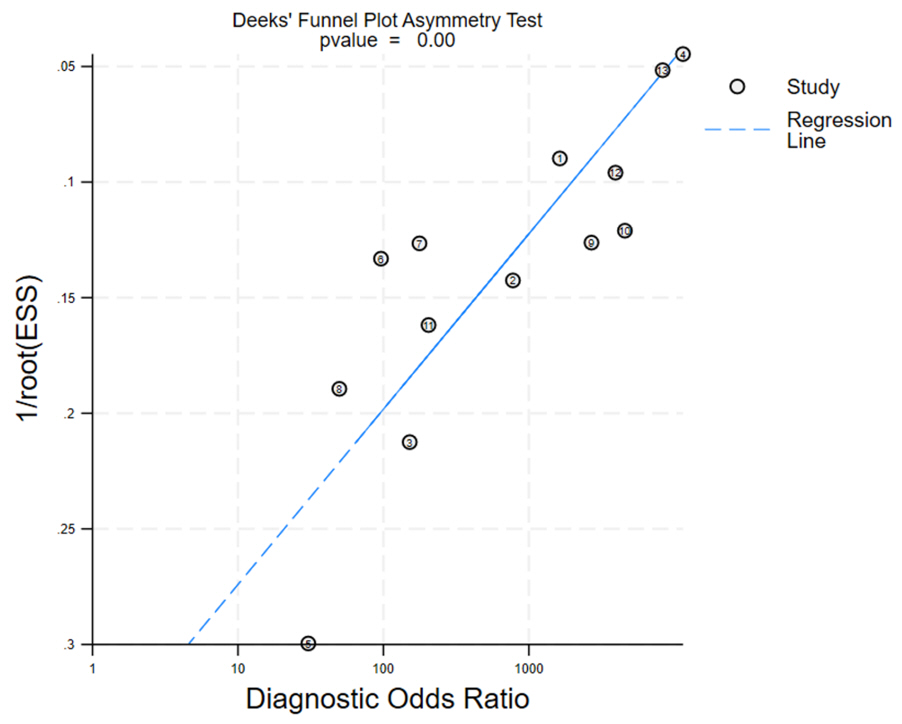

Supplement: Supplementary file 2 — Figure S2. Deek’s funnel plot asymmetry test of 13 included studies. [file CAM4-14-e70414-s001.jpg]
