# Supplementary material for: Diagnostic Performance and Safety of Ultrasound‐Guided Core Needle Biopsy for Diagnosing Lymphoma: A Systematic Review and Meta‐Analysis
Source: Cancer Med. 2025 Jan 6;14(1):e70414. doi: 10.1002/cam4.70414 (PMC11702417; doi:10.1002/cam4.70414)
Supplement: Supplementary file 3 — Table S1. Outcomes: complications of USCNB. [file CAM4-14-e70414-s003.docx]

**Supplementary table 1.** Outcomes: complications of USCNB.

| First author  (Year of  publication) | Complication Information | | | |
| --- | --- | --- | --- | --- |
|  | No. of complication  (Complication rate) | Pain | Bleeding or hematoma | Others |
| Adeel (2021) | 5 (1.7%) | 0 | 2 | 3  (2 vasovagal attacks,  1 transient facial weakness related to local anesthesia) |
| Allin (2017) | NA | NA | NA | NA |
| Baer (2021) | 0 (0%) | 0 | 0 | 0 |
| Cohen (2021) | 5 (13.9%) | 0 | 1 | 4  (bruising) |
| Elhamdoust (2020) | 2 (5%) | 2 | 0 | 2 |
| Groneck (2016) | 3 (2.2%) | 0 | 1 | 2  (1 self-limiting lymph fistula,  1 transient hypoesthesia of the trigeminal nerve) |
| Howlett (2006) | 0 (0%) | 0 | 0 | 0 |
| Kiliçarslan (2017) | NA | NA | NA | NA |
| Kim (2007) | 0 (0%) | 0 | 0 | 0 |
| Nguyen (2014) | 0 (0%) | 0 | 0 | 0 |
| Pfeiffer (2009) | 0 (0%) | 0 | 0 | 0 |
| Pugliese (2017) | 22 (11.9%) | 16 | 6 | 0 |
| Wilczynski (2020) | 11 (1.4%) | 0 | 11  (1 active bleeding due to venous malformation in cervical LN) | 0 |

**Supplementary Figure legends**

**Supplementary Figure 1.** Summary receiver operating characteristics (SROC) curves of US-guided CNB for diagnosis in lymphoma patients

**Supplementary Figure 2.** Deek’s funnel plot asymmetry test of 13 included studies
